# PI3K/AKT/mTOR signaling pathway: an important driver and therapeutic target in triple-negative breast cancer

**DOI:** 10.1007/s12282-024-01567-5

**Published:** 2024-04-17

**Authors:** Huan-ping Zhang, Rui-yuan Jiang, Jia-yu Zhu, Ke-na Sun, Yuan Huang, Huan-huan Zhou, Ya-bing Zheng, Xiao-jia Wang

**Affiliations:** 1 Zhejiang Cancer Hospital, Hangzhou Institute of Medicine (HIM), Chinese Academy of Sciences, Hangzhou, 310000 Zhejiang China; 2 Wenzhou Medical University, No. 270, Xueyuan West Road, Lucheng District, Wenzhou, 325027 Zhejiang China; 3 Zhejiang Chinese Medical University, No. 548, Binwen Road, Binjiang District, Hangzhou, 310000 Zhejiang China

**Keywords:** Triple-negative breast cancer, PI3K, AKT, PTEN, Targeted therapy

## Abstract

Triple-negative breast cancer (TNBC) is a highly heterogeneous tumor lacking estrogen receptor (ER), progesterone receptor (PR), and human epidermal growth factor receptor 2 (HER2) expression. It has higher aggressiveness and metastasis than other subtypes, with limited effective therapeutic strategies, leading to a poor prognosis. The phosphoinositide 3-kinase (PI3K)/protein kinase B (AKT)/mechanistic target of rapamycin (mTOR) signaling pathway is prevalently over-activated in human cancers and contributes to breast cancer (BC) growth, survival, proliferation, and angiogenesis, which could be an interesting therapeutic target. This review summarizes the PI3K/AKT/mTOR signaling pathway activation mechanism in TNBC and discusses the relationship between its activation and various TNBC subtypes. We also report the latest clinical studies on kinase inhibitors related to this pathway for treating TNBC. Our review discusses the issues that need to be addressed in the clinical application of these inhibitors.

## Introduction

Breast cancer (BC) is the most common and life-threatening malignancy affecting women, with approximately 2.3 million new cases and 685,000 deaths reported worldwide in 2020 [1]. Clinically, this heterogeneous disease is classified into three main types based on estrogen receptor (ER), progesterone receptor (PR), and human epidermal growth factor receptor 2 (HER2) status: hormone receptor (HR)-positive, HER2-positive, and triple-negative breast cancers (TNBC) [1]. Recent advances in endocrine and anti-HER2 therapies have improved the survival of patients with HR-positive and HER2-positive BC [1]. However, TNBC has a more aggressive and metastatic nature and a worse prognosis, and 46% of TNBC patients develop distant metastases, with a recurrence of 1.6–3.4 years [2]. Traditionally, TNBC was defined as a BC group lacking ER and PR expression, HER2 overexpression, or gene amplification. In 2011, Lyman et al. classified them into six subgroups: basal-like 1/2 (BL1/2), immunomodulatory (IM), mesenchymal (M), mesenchymal stem-like (MSL), and luminal androgen receptor (LAR) [3].

**Fig. 1:**
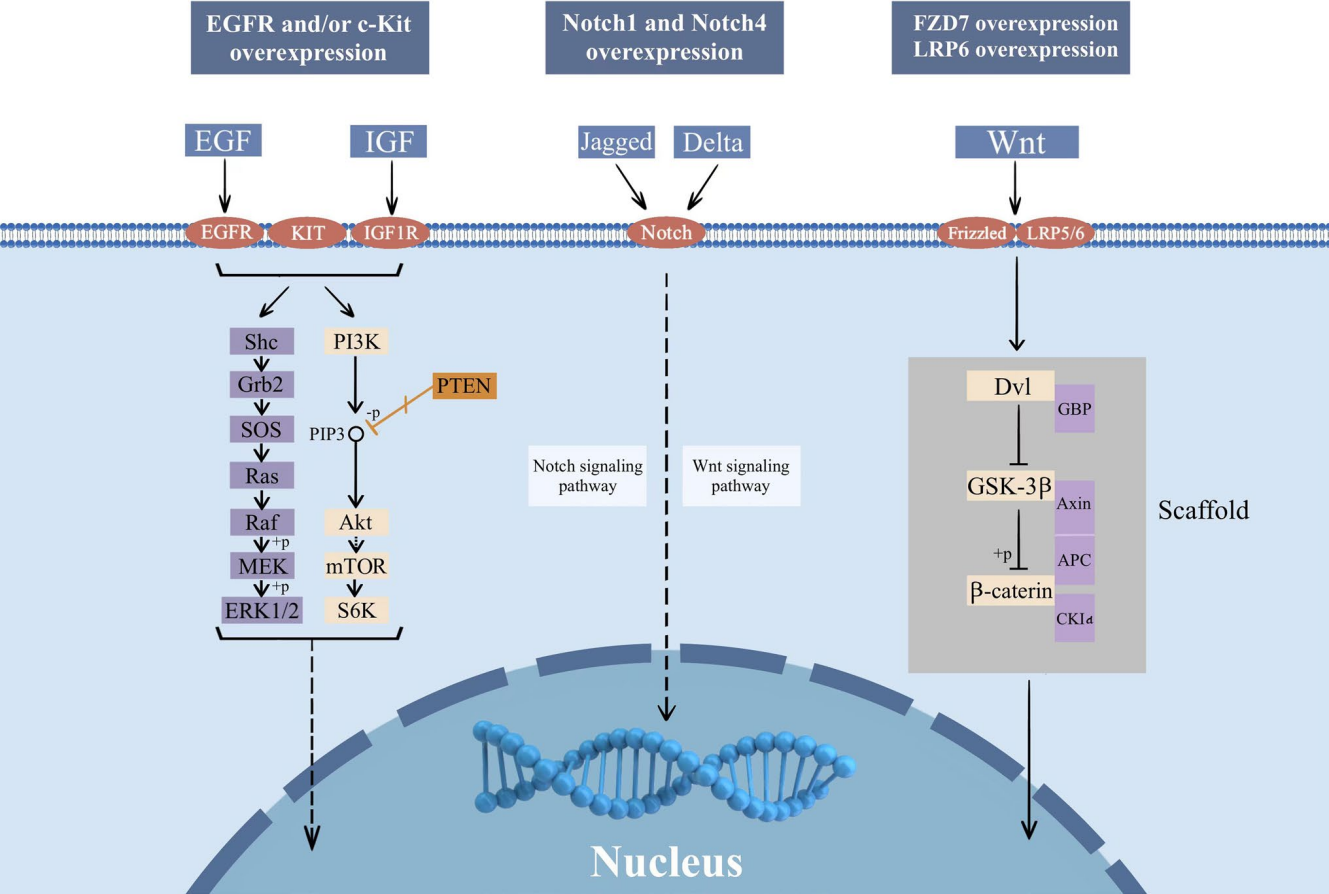
Major abnormal signaling pathways in TNBC. Although no clear driver gene has been found for TNBC at present, the occurrence and development of TNBC are closely related to the abnormalities of many signaling pathways. After NGS sequencing of many clinical samples, it was found that the EGFR signaling pathway, Notch signaling pathway, and Wnt signaling pathway were the most common abnormalities in patients with TNBC. This figure is drawn by AI

Non-selective chemotherapy—represented by anthracyclines and taxanes—is the traditional treatment option for patients with locally recurrent inoperable or metastatic triple-negative breast cancer (mTNBC). However, the efficacy of single or combination chemotherapy is poor, and the median overall survival (mOS) of patients with advanced TNBC rarely exceeds 12–18 months [3–5]. In addition, TNBC-targeted therapies include monoclonal antibody (mAb), antibody–drug conjugate (ADC), peptide–drug conjugate (PDC), and so on [6]. Recently, different immunotherapeutic modalities, including immune checkpoint blockade, vaccination, and adoptive cell transfer, have been extensively studied in the clinical setting of BC, particularly in patients with TNBC [7]. Several molecular pathways are activated in TNBC **(**Fig. 1**)**, particularly the phosphoinositide 3-kinase (PI3K)/protein kinase B (AKT)/mechanistic target of rapamycin (mTOR) signaling pathway—a key TNBC survival and resistance mechanism and a promising molecular target for treating TNBC [4].

**Fig. 2:**
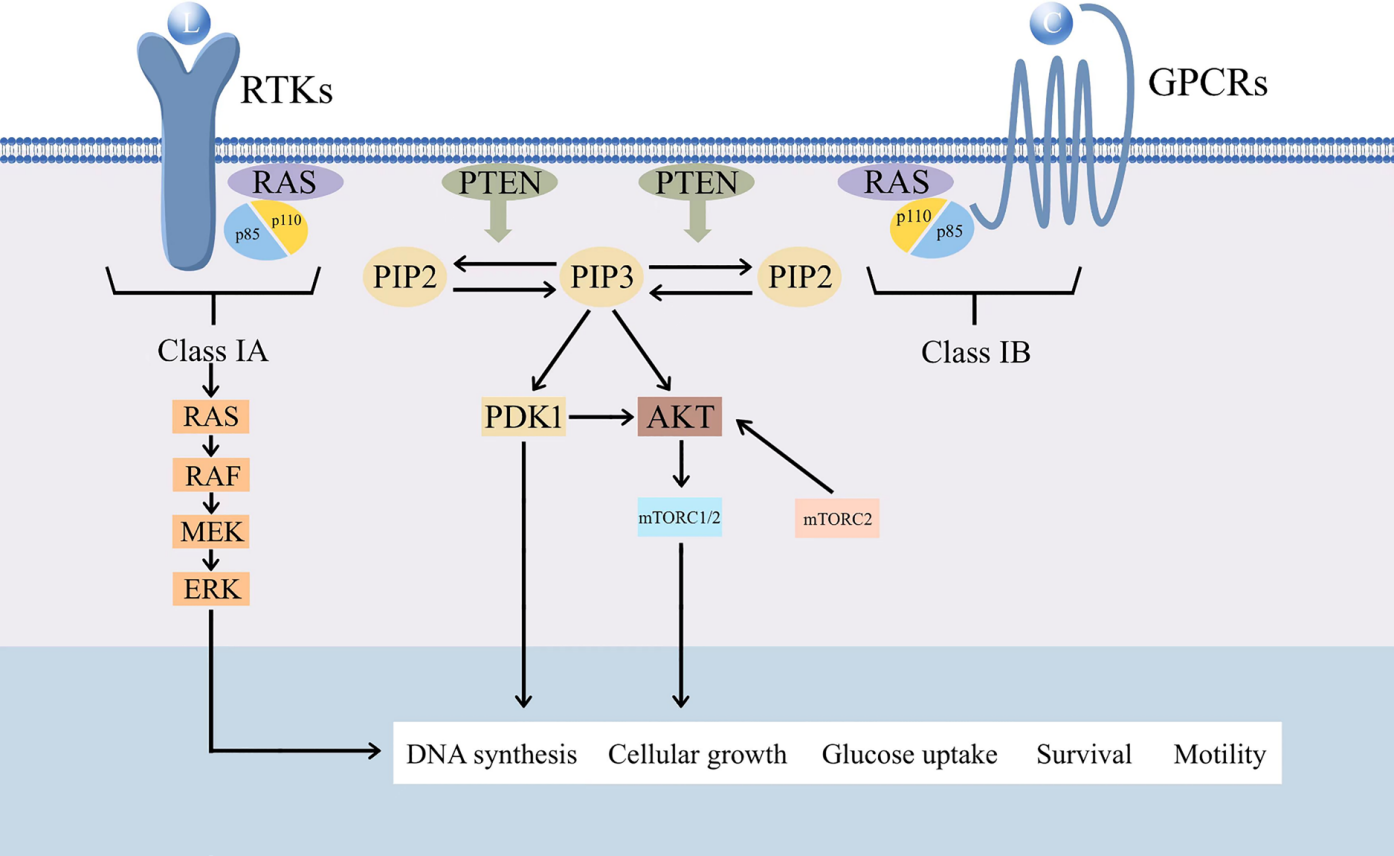
Summary diagram of the PI3K pathway and cellular activation pathways. PI3K is divided into three classes, of which class I can be subdivided into classes IA and IB. Class IA features tyrosine kinase receptors (RTKs), and class IB is associated with G-protein-coupled receptors (GPCRs). When the corresponding receptor is activated by a growth factor (L) or a chemokine (C), PI3K is recruited to the plasma membrane and is activated, leading to the phosphorylation of PIP2 to produce PIP3. PIP3 recruits and binds AKT and proteins with pleckstrin homology (PH) structural domains such as PDK1. Subsequently, AKT is phosphorylated and activated by PDK1 and the mammalian target of rapamycin complex 2 (mTORC2), triggering several phosphorylation-based signaling cascades. In addition, PTEN negatively regulates the activation of PIP3 by 3’-phosphatase activity, which converts PIP3 to PIP2, thereby stopping the phosphorylation cascade

The PI3K/AKT/mTOR signaling pathway—one of the most common over-activated pathways in human cancers—is abnormally altered in nearly 70% of BC [5]. This pathway links receptor tyrosine kinase (RTK) signaling to cell growth and survival regulation, and excessive activation can promote increased cell proliferation, inhibit apoptosis, and contribute to abnormal cell differentiation and autophagy, forming tumors and promoting metastasis [8]. PI3K is stimulated by activated RTK and phosphorylates Ptdlns-4,5-p2 (PIP2) to Ptdlns-3,4,5-p3 (PIP3) at the plasma membrane, initiating the PI3K pathway [9]. AKT and mTOR are key nodes in this pathway after PI3K activation. Sequential activation of these nodes promotes cellular proliferation, survival, and migration **(**Fig. 2**)**. Additionally, the PI3K pathway is regulated by several phosphatases, including phosphatase tensin homolog deleted on chromosome 10 (PTEN) and polyphosphate (5-phosphatases). PTEN negatively regulates PI3K signaling by dephosphorylating PIP3 to PIP2 and silencing AKT signaling [10]. PIP3 can be hydrolyzed by 5-phosphatase to produce PtdIns (3,4) P2, which can bind and activate pyruvate dehydrogenase kinase 1 (PDK1) and AKT, thereby activating the PI3K pathway [11].

Alterations or mutations in the PI3K/AKT/mTOR pathway occur in 25% of primary TNBC and possibly more frequently in mTNBC. Meanwhile, inhibitors associated with this pathway can significantly treat TNBC and are clinically useful. This review summarizes the PI3K/AKT/mTOR signaling pathway activation mechanism in TNBC and discusses the relationship between its activation and various TNBC subtypes. Our review reports the latest clinical studies on kinase inhibitors related to this pathway for TNBC treatment. We also discuss the issues that must be addressed in the clinical application of these inhibitors.

## Activation of the PI3K pathway in TNBC

### PIK3CA activating mutations

PI3Ks are intracellular signaling enzymes divided into three mammal classes [12]. Notably, class I PI3K is the predominant type driving tumorigenesis and has four catalytic isoforms, each comprising a regulatory subunit (p85α/β/γ) and a catalytic subunit (p110α/β/δ/γ) [13, 14]. The PIK3CA (PIK3CA encodes p110α) mutation is an oncogenic mechanism associated with PI3K pathway over-activation in BC, which over-activates p110α, enhances PIP2 phosphorylation, and increases PIP3 accumulation, resulting in sustained downstream pathway activation [14]. PIK3CA is the second most commonly mutated gene in TNBC, accounting for 9% of primary TNBC and possibly higher in advanced TNBC [15]. PIK3CA mutations modestly increase TNBC cell proliferation and significantly inhibit their apoptosis [16]. A study on the correlation between PI3K pathway activation and specific sites of BC metastasis revealed that the PIK3CA mutation rate was significantly higher in metastatic liver lesions than in other metastatic sites. This suggests that activation of the PI3K/AKT/mTOR pathway may represent an organ-specific drug target signaling for liver metastases in BC [17].

### AKT1 activating mutations

AKT, or protein kinase B, is a key effector molecule downstream of the PI3K pathway [14]. After PI3K is activated, accumulated PIP3 recruits intracellular PDK1 and AKT to the cell membrane. PDK1 phosphorylates Thr308 of AKT, and mTORC2 phosphorylates Ser473 of AKT, allowing AKT to be fully activated. Moreover, PDK1 indirectly enhanced mTORC2 activity. Activated AKT phosphorylates the most critical downstream effector, mTOR complex 1 (mTORC1), which promotes cell proliferation and oncogenic transformation [18]. AKT has three isoforms: AKT1/2/3. AKT gene amplification (common in AKT1) is more prevalent in BC [18], and 2.5% of AKT1 proteins have E17K mutations in the PHD structural region, resulting in aberrant AKT1 activation [19]. AKT1 promotes cell proliferation by upregulating S6 and cyclin D1 and inhibits cell migration and invasion. AKT2 promotes cell migration and invasion by inducing F-actin and waveform protein and is involved in distant dissemination. AKT3 is overexpressed in TNBC (14%) and promotes cell growth more than AKT1/2, but not invasion [20].

### mTOR activating mutations

mTOR is a serine/threonine protein kinase, of which mTORC1 is the main PI3K/AKT pathway effector [21]. mTORC1 is highly activated in cancer and promotes protein synthesis by phosphorylating p70S6 kinase 1 (S6K1) and eIF4E-binding protein (4EBP), and new lipid synthesis through sterol response element-binding protein (SREBP) [22]. Additionally, it promotes cell growth and division by promoting nucleotide production and inhibiting autophagy [23, 24]. Saxton et al. described these mechanisms [21]. Besides, mutations in the upstream tumor suppressors TP53 and LKB1 and downstream negative regulator TSC1/2 complexes activate mTOR [25, 26]. mTORC2 is involved in the composition of the actin cytoskeleton and regulates AKT phosphorylation [27]. The mTOR inhibitor everolimus has been approved for treating postmenopausal HR-positive and HER2-negative patients with advanced BC. The role of everolimus in TNBC is described in more detail later.

### PTEN inactivating mutations/loss

PTEN is an important tumor suppressor that dephosphorylates protein substrates on Tyr, Ser, and Thr phosphorylated peptides, thereby inactivating these substrates [28]. In the PI3K pathway, PTEN silences signaling by dephosphorylating PIP3 to PIP2, preventing AKT activation [29]. The tumor suppressor function of PTEN is influenced individually or synergistically by different mechanisms, including genetic alterations, transcriptional activation or repression, post-transcriptional regulation, and protein interactions [29]. In particular, after PTEN transcription, multiple mechanisms regulate its expression, one of which is noncoding RNAs, including microRNAs and competing endogenous RNAs (ceRNAs). MicroRNAs—one of the key regulators of PTEN—can inactivate PTEN and act as an oncogenic player [30]. In BC, miR-29b and miR-301, which are microRNAs targeting PTEN, inhibit PTEN protein levels, enhance cell proliferation, migration, and invasion, and promote tumor development [31, 32]. In addition, miR-498 is overexpressed in TNBC tissues and cell lines, reducing PTEN and activating PI3K-AKT signaling, which promotes TNBC cell proliferation and migration [33]. PTENP1 is a ceRNA with a sequence homologous to PTEN and can be used as a decoy to attract microRNAs targeting PTEN to prevent translation inhibition [34]. Additionally, post-translational protein modifications and interactions also can modulate PTEN activity [35].

## Association of TNBC subtypes with alterations in PI3K pathway

**Fig. 3:**
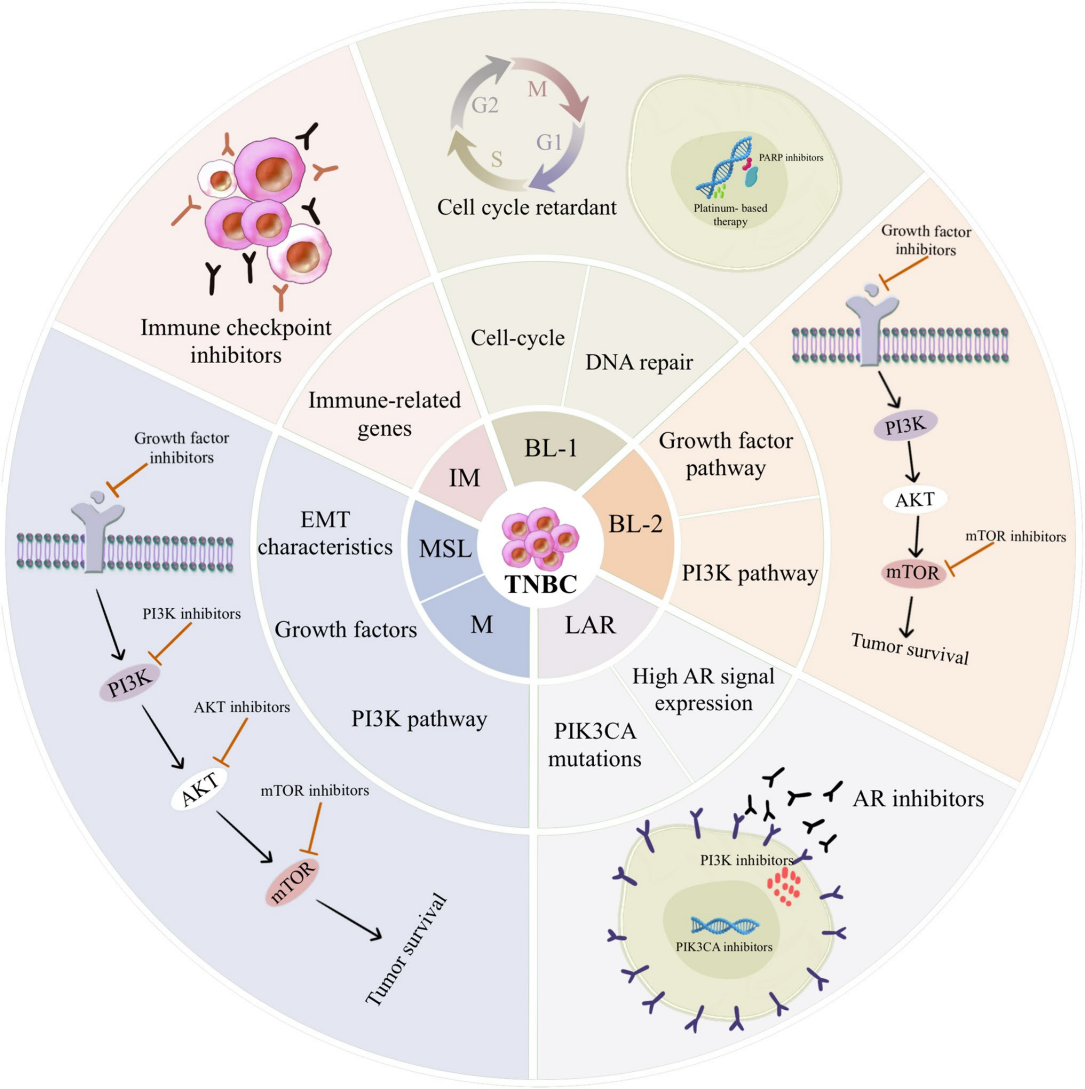
Characteristics and potential therapies based on the TNBC molecular subtype

In 2011, Lyman et al. classified TNBC into six subgroups, and genetic analysis revealed that BL1 is involved in the DNA damage response and cell cycle genes, whereas BL2 has articular and myoepithelial markers and is involved in the growth factor and PI3K pathways [36]. Two mesenchymal subtypes (M and MSL) are associated with EMT gene overexpression and growth factor signaling [36]. The M subtype is metaplastic breast cancer (MBC) because of its highly activated cell migration-related signaling pathways, extracellular matrix–receptor interaction, and differentiation pathways. Additionally, it has tissue features that are sarcoma-like or squamous epithelial cell-like and is easily resistant to chemotherapy [37]. mTOR inhibitors or drugs targeting EMT may be effective in the M subtype, and patients with the MSL subtype can be treated with PI3K inhibitors and anti-angiogenic drugs [36, 37]. The IM subtype has an abundance of immune cell-related genes and signaling pathways, such as the Th1/Th2, B cell receptor, and NK cell pathways, and can be optionally treated with immune checkpoint inhibitors [36, 38]. The LAR subtype has highly activated hormone-related signaling pathways, including steroid synthesis, androgen and estrogen metabolism, and porphyrin metabolism pathways. Significantly, the androgen receptor (AR) and many of its downstream metabolic markers and coactivators are detected in the LAR subtype, with mRNA levels of AR being nine times higher than other subtypes [39]. Therefore, anti-AR therapy is an option for patients with LAR subtype. Figure 3, drawn by AI, summarizes the characteristics and potential treatments for each subtype.

Based on genetic sequencing of TNBC cases in China in 2019, TNBC was classified into four subtypes by Jiang et al.: LAR, immunomodulatory, basal-like immune-suppressed, and mesenchymal-like [40]. When comparing previous data from the Cancer Genome Atlas (TCGA), they found an increased frequency of PIK3CA mutations in the LAR subtype [40]. Furthermore, TNBC cell lines contained PIK3CA gene mutations in 40% of LAR and 23% of MSL, while AKT1 mutations were more common in LAR than in other subtypes [41, 42]. In the basal-like subtype, PIK3CA and AKT1 mutations were rare, and PTEN protein expression was low compared with other subtypes with heterozygous loss of the PTEN copy number identified in 46.1% [41, 43].

## Clinical data of studies targeting PI3K pathway in TNBC

### PI3K inhibitors

PI3K inhibitors are divided into specific PI3K and pan-PI3K inhibitors. In BC, including TNBC, PIK3CA mutations are very common; consequently, PI3Kα-specific inhibitors—a class of selective oral inhibitors targeting the PI3K catalytic subunit P110α class I—have been extensively studied. They can also inhibit other subunits; however, all class members have a significantly reduced effect on PI3Kβ [44]. PI3Kα-specific inhibitors include alpelisib, taselisib, inavolisib, and serabelisib. The PI3Kγ inhibitor eganelisib and the dual PI3K and mTOR inhibitors gedatolisib have received attention. Pan-PI3K inhibitors can inhibit the kinase activity of all isoforms of class I PI3K: α, β, γ, and δ, including buparlisib, pictilisib, copanlisib, and others [45]. Several preclinical studies have depicted that PI3K inhibitors combined with chemotherapy, immunotherapy, AR, Poly (ADP ribose) polymerase (PARP), and CDK4/6 inhibitors may be new strategies for treating TNBC [42, 46, 47].

Alpelisib (BYL719) is an oral specific PI3Kα inhibitor and it effectively reduces the risk of treatment-related toxic side effects and extends the therapeutic window compared with pan-PI3K inhibitors [48]. Although alpelisib, combined with fulvestrant, was approved by the FDA for treating patients with HR-positive, HER2-negative, PIK3CA mutated advanced or progressive BC, its use in TNBC continues to be explored. A phase I/II trial that enrolled 42 patients with HER2-negative advanced-stage BC (including 12 TNBC patients) demonstrated that alpelisib combined with nab-paclitaxel had good tolerance and encouraging efficacy. Additionally, 40% of patients with PI3KCA mutations had longer PFS than the non-mutated group at 11.9 and 7.5 months, respectively [49]. Currently, EPIK-B3, an ongoing phase III, randomized, double-blinded, placebo-controlled trial, is testing the safety and efficacy of this combination in advanced TNBC patients carrying a PIK3CA mutation or PTEN deletion (ClinicalTrials.gov Identifier: NCT04251533). Additionally, two phase I trials evaluated the activity of buparlisib plus olaparib and alpelisib plus olaparib combinations in germline BRCA-mutant and wild-type recurrent BC (including TNBC patients) and ovarian cancer, respectively. This shows significant central nervous system toxicity in the former combination and favorable safety in the latter. These results provide preliminary clinical evidence for the synergy between PI3K and PARP inhibitors [50, 51]. Notably, a phase Ib clinical trial of alpelisib plus enzalutamide for treating AR and PTEN-positive metastatic BC patients is ongoing, which may provide clinical evidence for the combination of AR and PI3Kα inhibitors in managing advanced TNBC (ClinicalTrials.gov Identifier: NCT03207529) (Table 1).

**Table 1:** Clinical trials of the main PI3K inhibitors and AKT inhibitors in TNBC

Target	Drugs	Clinical trials	Phase	Intervention	Outcomes/primary endpoint	Status
PI3K	Alpelisib (BYL719)	NCT02379247	I/II	Nab-paclitaxel + alpelisib	Recommended phase II dose of alpelisib; ORR of the subject treated with phase II dose of alpelisib: 60% in the ER + population and 58% in the TNBC population	Completed [49]
		NCT01623349	Ib	Olaparib + alpelisib	Maximum tolerated and recommended phase II doses	Completed [51]
		NCT04251533 (EPIK-B3)	III	Nab-paclitaxel + alpelisib or placebo	PFS and ORR	Ongoing [/]
		NCT03207529	Ib	Enzalutamide + alpelisib	MTD	Ongoing [/]
	Taselisib (GDC-0032)	NCT02457910 (TBCRB 032)	Ib/II	Enzalutamide + taselisib vs. enzalutamide alone	16 weeks CBR: 35.7% vs. 0% (in patients receiving the combination vs. enzalutamide alone); 42.9% vs. 28.6% (in PIK3CA/AKT/mTOR mutated population vs. none); 75% vs.12.5% (in LAR subtype vs. all other subtypes)	Completed [53]
		NCT02389842 (PIPA trial)	Ib	Palbociclib + taselisib	Recommended dose for phase II, safety, and toxicity. Other findings in PIK3CA mutated; ER-population: ORR:10%, CBR: 30%, and mPFS: 3.6 months	Completed [55]
	Serabelisib (TAK-117)	NCT03193853	II	Tak-228 + serabelisib	ORR	Ongoing [/]
	Eganelisib (IPI-549)	NCT03961698 (MARIO-3)	II	Eganelisib + nab-paclitaxel + atezolizumab	ORR: 55.3% and DCR: 84.2% in ITT population; ORR: 66.7% and DCR: 91.7% in PD-L1-positive patients; ORR: 47.8% and DCR: 78.3% in PD-L1-negative patients	Ongoing [57]
		NCT03719326 (ARC-2)	I/Ib	Etrumadenant + pegylated liposomal doxorubicin (PLD) ± eganelisib	Safety and tolerability	Ongoing [/]
	Gedatolisib (PKI-587)	NCT01920061	Ib	Gedatolisib + docetaxel or cisplatin or dacomitinib	10.0% of the patients (7/70) had dose-limiting toxicities. gedatolisib + cisplatin in TNBC: ORR: 40% in first-line and 33.3% in second/third-line	Completed [59]
		NCT03243331	I	Gedatolisib + cofetuzumab pelidotin	Tolerability	Completed [58]
		NCT03911973	II	Talazoparib + gedatolisib	MTD and ORR	Ongoing [/]
	Menarini (MEN1611)	NCT05810870 (SABINA)	II	Menarini + eribulin; menarini monotherapy	Safety, tolerability, and efficacy	Ongoing [/]
	Buparlisib (BKM120)	NCT01572727 (BELLE-4)	II/III	Buparlisib + paclitaxel vs placebo + paclitaxel	mPFS in full: 8.0 vs. 9.2 months; mPFS in PI3K pathway-activated population: 9.1 vs. 9.2 months; Phase III trial not initiated due to ineffectiveness	Completed [64]
		NCT01790932	II	Buparlisib monotherapy	mPFS: 1.8 months; mOS: 11.2 months; CBR: 12%; adverse events: fatigue, nausea, hyperglycemia, anorexia, depression, and anxiety	Completed [63]
	Copanlisib (BAY80–6946)	NCT04345913	I/II	Copanlisib + eribulin	Safety and efficacy	Ongoing [/]
AKT	Capivasertib (AZD5363)	NCT02423603 (PAKT)	II	Paclitaxel + capivasertib vs Paclitaxel + placebo	mPFS: 5.9 vs. 4.2 months in the ITT populations; 9.3 vs. 3.7 months in the PIK3CA/AKT/PTEN mutated population. OS: 19.1 vs.13.5 months in ITT populations, but insignificantly different	Completed [66, 67]
		NCT03997123 (CAPitello290)	III	Paclitaxel + capivasertib vs paclitaxel + placebo	OS	Ongoing [/]
		NCT03742102 (Begonia)	Ib/II	Durvalumab + paclitaxel + capivasertib	Safety	Ongoing [/]
	Ipatasertib (GDC-0068)	NCT02162719 (LOTUS)	II	Paclitaxel + ipatasertib vs paclitaxel + placebo	PFS in ITT population: 6.2 vs. 4.9 months; PFS in PTEN-low: 6.2 vs. 3.7 months; PFS in PIK3CA/AKT1/PTEN-mutated patients: 9.0 vs. 4.9 months; mOS in the final analysis: 25.8 vs. 16.9 months	Completed [69, 70]
		NCT03337724 (Ipatunity130)	III	Paclitaxel + ipatasertib vs paclitaxel + placebo	No improvement in PFS in TNBC patients with PIK3CA/AKT1/PTEN mutations	Completed [71]
		NCT04464174 (PathFinder)	IIa	Ipatasertib + eribulin vs ipatasertib + capecitabine vs ipatasertib + carboplatin + gemcitabine	Safety and tolerability	Ongoing [/]
		NCT04177108 (IpaTunity170)	III	Paclitaxel ± atezolizumab ± ipatasertib	PFS and OS	Ongoing [/]
		NCT03853707	I/Ib	Ipatasertib + carboplatin + paclitaxel vs. ipatasertib + carboplatin vs ipatasertib + capecitabine + atezolizumab	Confirm the recommended phase II dose (RPIID) and obtain evidence of this activity	Ongoing [/]
		NCT03800836	Ib	Ipatasertib + atezolizumab + paclitaxel/nab-paclitaxel/doxorubicin and cyclophosphamide (AC)	Safety and tolerability	Ongoing [/]

Taselisib (GDC-0032) is another PI3Kα inhibitor; besides selective inhibition of P110α, its effect mechanism includes a proteasome-mediated degradation specific to the mutant oncoprotein [52]. A multi-institutional phase Ib/II study, TBCRC0322, evaluating the safety and efficacy of taselisib plus enzalutamide, displayed that the combination was tolerated [53]. However, the SANDPIPER trial demonstrated limited benefit of taselisib in metastatic BC, and drug development was halted, resulting in a portion of the phase II trial not being completed [54]. In 17 patients with metastatic AR+ ($\ge$ 10%) TNBC, the clinical benefit rate (CBR) was 35.7% in the combination group, while none of the patients on enzalutamide alone benefited. The CBR between PIK3CA mutated and unmutated groups was non-significant and higher in patients with the LAR subtype than other subtypes (75% vs. 12.5%, *p* = 0.06). Another phase Ib trial investigated the safety and efficacy of a combination of the CDK4/6 inhibitors palbociclib and taselisib in solid tumors, including the TNBC cohort. In patients with PIK3CA mutations and ER-advanced BC (eight TNBC and two HER2-positive), ORR, CBR, and mPFS were 10%, 30%, and 3.6 months, respectively [55].

Inavolisib (GDC-0077) is a recently developed strong p110α inhibitor that induces specific degradation of the mutated form of PIK3CA. Several relevant phase II/III clinical studies (ClinicalTrials.gov Identifiers: NCT05306041, NCT05646862, NCT04191499, and NCT05894239) have been conducted in metastatic and advanced BC patients with PIK3CA mutations and HR-positive or HER2-positive phenotypes, but have not been studied in TNBC. Serabelisib (TAK-117) is a novel PI3K inhibitor with high selectivity for p110α and a strong ability to induce cell proliferation and inhibit apoptosis. A phase II clinical trial evaluated the combination of TAK-228 and TAK-117 for treating metastatic TNBC (ClinicalTrials.gov Identifier: NCT03193853).

Eganelisib (IPI-549) is a highly selective PI3Kγ inhibitor (≥ 150-fold compared with class I PI3K isoforms and other kinases) with anti-tumor activity alone and has shown feasibility when combined with a programmed cell death 1/programmed cell death ligand 1 (PD1/PDL1) inhibitor in preclinical studies. The MARIO-1 trial—a first-in-human phase I/Ib trial—evaluated the safety and efficacy of eganelisib monotherapy or in combination with nivolumab in patients with solid tumors. The trial revealed that the most common grade ≥ 3 toxicities associated with eganelisib monotherapy were increased levels of alanine aminotransferase (18%), aspartate aminotransferase (18%), and alkaline phosphatase (5%). Based on the trial results, 30 or 40 mg of eganelisib once daily combined with a PD1/PDL1 inhibitor would be more appropriate for a phase II study [56]. MARIO-3 is a phase II multi-arm cohort study; cohort A assessed the effectiveness of eganelisib combined with albumin paclitaxel and atelizumab in patients with advanced or metastatic TNBC, where cohort A1 was PDL1-positive and cohort A2 was negative (ClinicalTrials.gov Identifier: NCT03961698). The latest results at the 2022 San Antonio Breast Cancer Symposium indicate that this triple combination therapy has promising anti-tumor activity (ORR 55.3% and DCR 84.2%) and manageable toxicity regardless of PD-L1 status [57].

Gedatolisib (PKI-587) is a potent reversible dual inhibitor that selectively targets all class I PI3K isoforms and mTOR. A phase I trial enrolled 18 patients with advanced TNBC to investigate the safety of gedatolisib combined with cofetuzumab pelidotin, demonstrating that this combination had a good safety profile and promising clinical activity, which warrants further investigation to treat metastatic TNBC [58]. In another phase of the Ib trial, 107 patients with BC were treated with gedatolisib combined with docetaxel, cisplatin, or dacomitinib; 10% of evaluable patients (7/70) experienced dose-limiting toxicity, and the most common was grade 3 oral mucositis. Twenty-two patients with TNBC were treated with gedatolisib combined with cisplatin, with an ORR of 40% in the first line and 33.3% in the second/third line [59]. Currently, a phase II trial tests the effectiveness of gedatolisib plus talazoparib in advanced TNBC or BRCA1/2 mutated, HER2-negative BC (ClinicalTrials.gov Identifier: NCT03911973).

Menarini (MEN1611) is a novel oral PI3Kδ inhibitor that exhibits lower cytotoxic activity than taselisib in a p110δ-driven HER2-positive BC cell model and higher cytotoxic activity than alpelisib in a p110β-driven cellular model [60]. SABINA is an ongoing multicenter, double-cohort, non-comparative, open-label phase II clinical trial that aims to analyze the safety and efficacy of MEN1611, both as a monotherapy and in combination with eribulin, for treating locally advanced or metastatic TNBC with PIK3CA/PTEN mutations (ClinicalTrials.gov Identifier: NCT05810870).

Buparlisib (BKM120) is an oral pan-class I PI3K inhibitor that targets all PI3K isoforms. Two phase III clinical studies—BELLE-2 and BELLE-3—demonstrated the effectiveness of buparlisib plus fulvestrant in endocrine-resistant hormone-positive BC [61, 62]. However, BELLE-4 and an additional single-arm phase II clinical study demonstrated no clinical benefit of buparlisib in combination with paclitaxel or monotherapy in locally advanced or metastatic TNBC [63, 64]. Buparlisib has been discontinued in BC due to its serious adverse effects and poor efficacy. Pictilisib (GDC-0941) and copanlisib (BAY80–6946) are both pan inhibitors but have no clinical results in TNBC. A phase I/II trial is ongoing to evaluate the safety and efficacy of copanlisib in patients with metastatic TNBC (ClinicalTrials.gov Identifier: NCT04345913).

### AKT inhibitors

Capivasertib (AZD5363) is an orally administered, highly selective pan-AKT inhibitor with similar activity against AKT1/2/3 [65]. The PAKT trial was a randomized, double-blinded, placebo-controlled phase II clinical trial that recruited 140 patients with untreated metastatic TNBC. The trial aimed to assess the safety and efficacy of adding capivasertib to paclitaxel as a first-line treatment for TNBC patients. The results demonstrated that the median PFS in the capivasertib group increased from 4.2 to 5.9 months compared with the placebo group. In 28 patients with PIK3CA/AKT1/PTEN alteration, mPFS was 9.3 months in the capivasertib group vs. 3.7 months in the placebo group. These results suggest that adding the AKT inhibitor capivasertib to the first-line treatment of TNBC significantly prolongs PFS, with the benefit being more pronounced in patients with PIK3CA/AKT1/PTEN alterations. The final results of this trial showed that the capivasertib group had a longer OS than the placebo group (19.1 vs. 13.5 months; HR, 0.70), but the difference was not significant. However, contrary to previously published results, there was no difference in the clinical benefit between patients with or without alterations in PIK3CA/AKT1/PTEN. Herein, the most common adverse events were diarrhea (13% vs. 1%), infection (4% vs. 1%), rash (4% vs. 0%), and fatigue (4% vs. 0%), with equal proportions in both neutropenic groups (3%) [66, 67]. The safety and efficacy of capivasertib combined with paclitaxel as first-line treatment for mTNBC were evaluated in the phase III clinical trial CAPitello290 (ClinicalTrials.gov Identifier: NCT03997123). Additionally, the Begonia trial explores the efficacy and safety of durvalumab (MEDI4736) in combination with new oncology therapies for treating first-line metastatic TNBC, with trial group II investigating capivasertib in association with paclitaxel and durvalumab (ClinicalTrials.gov Identifier: NCT03742102).

Ipataserti (GDC-0068) is a highly selective ATP-competitive small AKT inhibitor that exhibits activity in various cancer cell lines and xenograft models, including BC [68]. The LOTUS trial evaluated the safety and efficacy of adding ipatasertib to the late first-line treatment of TNBC. Compared to the paclitaxel combined with placebo group, the results displayed an increase in PFS with the addition of ipatasertib to paclitaxel, and the PFS increased from 4.9 to 6.2 months in the ITT population and from 3.7 to 6.2 months in the low PTEN subgroup. Further analysis of 42 patients with PIK3CA/AKT1/PTEN-mutated demonstrated an even more significant improvement in their PFS, from 4.9 to 9.0 months. In the final analysis, the median OS was longer in the trial group than the placebo group, at 25.8 and 16.9 months, respectively. In all biomarker-defined subgroups (PTEN normal or low, PIK3CA/AKT1/PTEN altered or unaltered), median OS favored ipatasertib–paclitaxel. Unfortunately, patients carrying PI3K/AKT/mTOR mutations depicted no enhanced efficacy with paclitaxel plus ipatasertib [69, 70]. However, the phase III clinical trial IPATunity130 displayed the opposite results to LOTUS, demonstrating that paclitaxel combined with ipatasertib failed to improve PFS in TNBC patients with PIK3CA/AKT1/PTEN mutations [71]. Additionally, a multicenter, three-arm, phase II clinical study called PathFinder was designed to investigate the safety and efficacy of ipatasertib in combination with capecitabine, eribulin, or carboplatin plus gemcitabine in patients with unresectable locally advanced or metastatic TNBC (ClinicalTrials.gov Identifier: NCT04464174). PTEN loss and PI3K/AKT activation are the mechanisms of immunotherapy resistance in patients with TNBC [72]. AKT inhibitors are under development in clinical trials as potential strategies to enhance the efficacy of immunotherapy for TNBC. A phase III, double-blinded, placebo-controlled clinical trial, IpaTunity170, demonstrated an ORR of 54% for triple combination therapy (ipatasertib combined with paclitaxel/nab-paclitaxel and atezolizumab) in mTNBC (ClinicalTrials.gov Identifier: NCT04177108) [73]. However, the BARBICAN trial indicated that this combination did not improve the clinical outcomes of neoadjuvant treatment [74]. Additionally, two phase I clinical trials are underway to explore the safety and efficacy of ipatasertib and atezolizumab combined with chemotherapy in metastatic TNBC (ClinicalTrials.gov Identifiers: NCT03853707 and NCT03800836).

### mTOR inhibitors

The mTOR inhibitors include everolimus and temsirolimus. The BOLERO-2, PrE0102, and GINECO studies demonstrated that everolimus plus endocrine therapy significantly prolonged PFS in postmenopausal HR-positive and HER2-negative advanced BC patients who failed endocrine therapy [75–77]. Meanwhile, BOLERO-4 and BOLERO-5 demonstrated that everolimus, in combination with letrozole and exemestane, respectively, prolonged PFS in this group of patients [78, 79]. And the MIRACLE study depicted that everolimus plus endocrine therapy was effective in patients with premenopausal HR-positive and HER2-negative advanced BC [80]. Additionally, in a phase I clinical trial, the mTOR inhibitors temsirolimus and everolimus combined with the chemotherapeutic drug liposomal doxorubicin and the anti-angiogenic agent bevacizumab were examined in 52 metaplastic TNBC patients. Although the study reported a promising ORR (21%), the clinical trials have stopped [81].

## Conclusion

The PI3K/AKT/mTOR signaling pathway is crucial for TNBC cell growth, survival, proliferation, and angiogenesis, making it an important TNBC therapy target. PI3K (alpelisib) and mTOR (everolimus) inhibitors, which have FDA approval, provide more treatment options for advanced BC patients with HR-positive and HER2-negative status after progression on endocrine therapy; they have shown better ORR rates in TNBC clinical trials. Additionally, increasing PI3K/AKT/mTOR signaling pathway-related inhibitors has demonstrated safety and efficacy in various clinical studies, and their combination with conventional chemotherapy significantly prolonged PFS and OS. PI3K inhibitors, including pan and selective inhibitors, are important. Pan inhibitors have not yet illustrated the expected efficacy, but they have opened the way for specific PI3K α-specific inhibitors. Although PI3K-selective inhibitors are not licensed in the TNBC field, they have a wide development field and great potential for clinical application.

Most completed and ongoing clinical studies have been conducted with inhibitors of the PI3K signaling pathway in combination with taxane, including nab-paclitaxel, paclitaxel, and docetaxel. The PAKT and LOTUS trials showed that combining the AKT inhibitor with paclitaxel in advanced TNBC patients significantly prolonged PFS, especially in those with PI3K signaling pathway mutations. In addition, current clinical trials, which addressed PI3K inhibitors combined with AR, PARP, CDK4/6 inhibitors and immunotherapy, have shown some efficacy. Consequently, the subsequent clinical study design could be based on PI3K/AKT/mTOR signaling pathway-related inhibitors combined with chemotherapy as the cornerstone, with the option of combining AR, PARP, and CDK4/6 inhibitors and immunotherapy to manage TNBC. Furthermore, developing specific treatment regimens based on the features of each TNBC subtype requires further exploration.

However, PI3K signaling pathway inhibitors still face many challenges for further clinical applications. First, although PI3K plays a central role in oncogenesis, only modest anti-tumor activity has been observed, and the future of PI3K signaling pathway inhibitors depends on the correct choice of combination therapy. Second, the toxic reactions associated with these inhibitors, such as fever, rash, pruritus, hyperglycemia, and mucositis, should not be ignored in clinical applications. Learning how to manage these drugs to improve patient compliance and researching more selective inhibitors are new challenges that must be addressed. The PI3K signaling pathway is vital for normal human cells; it responds to insulin and insulin-like growth factors and amino-acid-nutrients, regulates blood glucose and amino acids, and influences cellular autophagy. Consequently, we need to identify specific inhibitors of the PI3K signaling pathway associated with BC. Finally, there remains an incomplete understanding of the action and regulatory mechanisms of the PI3K signaling pathway, and the specific efficacy still needs to be confirmed by clinical trials and basic experimental studies.

## Data Availability

Not applicable.
